# Antiviral Susceptibility of Influenza A(H5N1) Clade 2.3.2.1c and 2.3.4.4b Viruses from Humans, 2023–2024

**DOI:** 10.3201/eid3104.241820

**Published:** 2025-04

**Authors:** Philippe Noriel Q. Pascua, Anton Chesnokov, Ha T. Nguyen, Han Di, Juan De La Cruz, Yunho Jang, Andrei A. Ivashchenko, Alexandre V. Ivachtchenko, Erik A. Karlsson, Borann Sar, Chin Savuth, Timothy M. Uyeki, Charles Todd Davis, Larisa V. Gubareva

**Affiliations:** Centers for Disease Control and Prevention, Atlanta, Georgia, USA (P.N.Q. Pascua, A. Chesnokov, H.T. Nguyen, H. Di, J.D.L. Cruz, Y. Jang, T.M. Uyeki, C.T. Davis, L.V. Gubareva); ChemDiv, San Diego, California, USA (A.A. Ivashchenko, A.V. Ivachtchenko); Avisa LLC, Hallandale Beach, Florida, USA (A.V. Ivachtchenko); Virology Unit, Institut Pasteur du Cambodge, Phnom Penh, Cambodia (E.A. Karlsson); US Centers for Disease Control and Prevention Country Office, Phnom Penh (B. Sar); National Institute of Public Health, Ministry of Health, Phnom Penh (C. Savuth)

**Keywords:** influenza A(H5N1), viruses, zoonoses, zoonotic infections, influenza, antiviral, drug resistance, polymerase inhibitors, antimicrobial resistance, respiratory infections

## Abstract

During 2023–2024, highly pathogenic avian influenza A(H5N1) viruses from clade 2.3.2.1c caused human infections in Cambodia and from clade 2.3.4.4b caused human infections in the Americas. We assessed the susceptibility of those viruses to approved and investigational antiviral drugs. Except for 2 viruses isolated from Cambodia, all viruses were susceptible to M2 ion channel-blockers in cell culture-based assays. In the neuraminidase inhibition assay, all viruses displayed susceptibility to neuraminidase inhibitor antiviral drugs oseltamivir, zanamivir, peramivir, laninamivir, and AV5080. Oseltamivir was ≈4-fold less potent at inhibiting the neuraminidase activity of clade 2.3.4.4b than clade 2.3.2.1c viruses. All viruses were susceptible to polymerase inhibitors baloxavir and tivoxavir and to polymerase basic 2 inhibitor pimodivir with 50% effective concentrations in low nanomolar ranges. Because drug-resistant viruses can emerge spontaneously or by reassortment, close monitoring of antiviral susceptibility of H5N1 viruses collected from animals and humans by using sequence-based analysis supplemented with phenotypic testing is essential.

During 2023–2024, human cases of highly pathogenic avian influenza (HPAI) A(H5N1) detected in Cambodia were caused by clade 2.3.2.1c viruses and in the United States were caused by clade 2.3.4.4b viruses (*1*; J. Siegers, unpub. data, https://doi.org/10.1101/2024.11.04.24313747). Except for 2 viruses from early 2023, clade 2.3.2.1c viruses contain most internal gene segments from clade 2.3.4.4b viruses, including polymerase acidic (PA), polymerase basic 2 (PB2), and matrix (M). Evidence of reassortment between the clades has been reported in viruses circulating in birds in Cambodia, Laos, and Vietnam (J. Siegers, unpub. data, https://doi.org/10.1101/2024.11.04.24313747).

The intercontinental spread of clade 2.3.4.4b viruses from Eurasia to North America that occurred in late 2021 was followed by a spread to Central and South America, causing devastating outbreaks in wild birds and domestic poultry and spilling over into a variety of mammals (*2*–*4*). Those viruses reassorted with other avian influenza A viruses to generate various genotypes of HPAI H5N1 viruses (*5*). Sporadic human cases were reported in Ecuador, Chile, and the United States during 2022–2023 (*6*–*8*). In March 2024, dairy cattle in Texas, USA, tested positive for clade 2.3.4.4b HPAI H5N1 virus belonging to genotype B3.13; detections at dairy farms in 14 additional US states followed (*9*). During March 28–September 30, 2024, a total of 15 viruses of the B3.13 genotype were detected in dairy and poultry farm workers in Texas (n = 1), Michigan (n = 2), Colorado (n = 10), and California (n = 1); an additional HPAI H5N1 virus was detected in a patient from Missouri who reportedly had no known animal exposure (Appendix 1 Table 1) (*10*–*13*).

The pandemic potential posed by HPAI H5N1 viruses necessitates close monitoring of their spread and evolution. Antigenic and antiviral testing are integral components of virologic surveillance and generate data that are used for biological risk assessment and decision-making regarding vaccines and therapeutics (*14*,*15*). Influenza antiviral drugs, prescribed as therapeutic or postexposure prophylactic agents, are needed to control influenza infections, especially when vaccines against novel influenza A virus are not available (*16*). However, resistance to antiviral drugs can emerge because of spontaneous mutation, gene reassortment, or selective pressure from exposure to the drugs and is a public health concern. 

Many countries have approved 3 classes of antiviral drugs to control influenza. The oldest are the M2 ion-channel protein blockers (M2 blockers) of influenza A viruses, amantadine and rimantadine. Substitutions at 5 residues within the M2 protein transmembrane domain confer cross-resistance to M2 blockers (*17*). Resistance to M2 blockers was also detected in some avian and swine viruses, including H5N1 (*18*,*19*).

Neuraminidase (NA) inhibitors are active against influenza A and B viruses, which require NA enzyme activity for efficient replication and transmission. This class includes oral oseltamivir, inhaled zanamivir and laninamivir (approved only in Japan), and intravenous peramivir. Among the NA inhibitors, oseltamivir is the most widely used influenza antiviral (*16*,*20*). Of note, most seasonal influenza A(H1N1) viruses circulating globally among persons during 2008–2009 were resistant to oseltamivir because of the resistance-conferring mutation NA-H275Y (N1 numbering). Oseltamivir-resistant influenza A(H1N1)pdm09 (pH1N1) viruses with this mutation were also detected in circulation and associated with clusters in several countries (*21*). In addition, NA-H275Y was sporadically detected in various other N1 subtype viruses, including H5N1 (*22*). Other NA mutations are associated with reduced inhibition by NA inhibitors, but their effect on drug phenotype is unknown. In recent years, detection of influenza viruses with such NA mutations, including NA-H275Y, were reported at low frequencies (<2%) (*23*). The emergence of viruses with resistance-conferring mutations and the limited antiviral options spurred the search for novel compounds with improved antiviral activity or different mechanisms of action. Accordingly, an investigational oral NA inhibitor, AV5080, was shown to potently inhibit NA activity of a diverse set of influenza viruses, including those with NA-H275Y (*24*).

Baloxavir targets the cap-dependent endonuclease activity of the (PA) protein and exhibits broad activity against influenza viruses of types A, B, C, and D (*25*). Viruses with reduced susceptibility to baloxavir are rare, but several PA mutations are implicated in treatment-emergent resistance, PA-I38T being the most common (*23*). PA inhibitors under investigation include tivoxavir (AV5116) (*26*). The cap binding activity of the PB2 protein is also an attractive target (*27*). The PB2 inhibitor pimodivir was shown to be active against influenza A viruses, including HPAI H5N1 viruses (*18*).

Antiviral susceptibility assessment of clade 2.3.4.4b HPAI H5N1 viruses collected from birds and mammals during 2021–2023 (*22*,*28*) revealed sporadic detection of drug-resistant viruses, some with novel mutations. A similar assessment of clade 2.3.2.1c viruses has not yet been reported. In this study, we conducted a comprehensive antiviral susceptibility assessment of H5N1 viruses from clades 2.3.2.1c and 2.3.4.4b isolated from humans in Cambodia, Chile, and the United States during 2023–2024. Analyses in this study have been approved by the Cambodian National Ethics Committee for Health Research (ethics approval no. 365NECHR/2024).

## Material and Methods

### Antiviral Compounds

We dissolved the M2 blockers, amantadine hydrochloride (Sigma-Aldrich, https://www.sigmaaldrich.com), rimantadine (Roche, https://www.roche.com), and the NA inhibitors oseltamivir carboxylate, zanamivir, peramivir, and laninamivir (BioSynth, https://www.biosynth.com) individually in sterile distilled water. We dissolved the PA inhibitor baloxavir acid (MedChem Express, https://www.medchemexpress.com) in DMSO (Sigma-Aldrich). We purchased the investigational drug pimodivir from MedChem Express; AY8050 and tivoxavir were provided by ChemDiv (https://www.chemdiv.com).

### Viruses

We propagated HPAI H5N1 viruses in 10-day old embryonated chicken eggs or in MDCK cells (American Type Culture Collection, https://www.atcc.org). We used the clade 2.3.4.4b HPAI H5N1 virus A/bald eagle/Florida/W22–134-OP/2022 (eagle/FL/22) and representative seasonal influenza (A and B) viruses as controls in phenotypic assays. We conducted handling and experiments with HPAI H5N1 viruses in an enhanced Biosafety Level 3 containment facility.

### Next-Generation Sequencing Analysis

We generated sequences by using a next generation-sequencing (NGS) platform (Illumina, https://www.illumina.com), analyzed by the iterative refinement meta-assembler (*29*) and aligned with sequences available from GISAID (https://www.gisaid.org) by using MAFFT version 7 (*30*). We deposited all sequences into GISAID.

### Virus Yield Reduction

We used a conventional yield reduction assay (*31*) to assess virus susceptibility to M2 blockers. We inoculated confluent monolayers of MDCK-SIAT1 cells with virus, then added growth medium containing antiviral and incubated at 37°C. After 16 or 21 hours postinfection (hpi), we collected supernatants, determined virus titers, and expressed them as 50% tissue-culture infectious dose (TCID_50_) per milliliter, according to the Spearman-Kaerber method.

### Neuraminidase Inhibition Assay

We assessed susceptibility to NA inhibitors by using the NA-Fluor Influenza Neuraminidase Assay Kit (Applied Biosystems, https://www.thermofisher.com) (*32*). We preincubated normalized virus preparations with the NA inhibitors for 45 minutes, followed by a 1-hour incubation with 2-(4-(methylumbelliferyl)-a-D-N-acetylneuraminic acid (Sigma-Aldrich) substrate. We measured fluorescence by using Cytation 7 (Agilent, https://www.agilent.com). We calculated the drug concentration required to inhibit 50% NA activity (IC_50_) on the basis of >3 independent tests.

### Influenza Replication Inhibition NA-Based Assay to Assess Susceptibility to M2 Blockers and Polymerase Inhibitors

We assessed susceptibility to M2 blockers and polymerase inhibitors by using the cell culture-based influenza replication inhibition NA-based assay (IRINA), as previously described (*33*). We added MDCK-SIAT1 cell suspension, serially diluted antiviral, and normalized virus preparations to 96-well microplates (Agilent). We then incubated the microplates at 37°C for 7 hours. We aspirated the supernatant and replaced it with 2-(4-(methylumbelliferyl)-a-D-N-acetylneuraminic acid substrate. After 1 hour of incubation at 37°C, the reaction was stopped, and we determined virus replication by measuring fluorescence by using Cytation 7 (Agilent). We determined the 50% effective concentration (EC_50_) values by using nonlinear regression (*33*).

## Results

### Susceptibility to M2-Blockers

During January 2023–September 2024, a total of 16 clade 2.3.2.1c HPAI H5N1 viruses in Cambodia and 15 clade 2.3.4.4b HPAI H5N1 viruses in the United States were detected in humans (Appendix 1 Table 1). By using available data, sequence analysis of those viruses and that of a 2.3.4.4b virus from a patient in Chile (Chile/23) revealed that they lacked molecular markers of resistance to M2 blockers, except for the 2 clade 2.3.2.1c viruses collected in early 2023 that shared M2-S31N (Tables 1, 2).

**Table 1:** M2 blocker susceptibility of highly pathogenic avian influenza A(H5N1) viruses isolated from humans in virus yield reduction assay, 2023–2024*

Influenza A(H5N1) virus	21 hpi					16 hpi			
	Virus titer†	log reduction in titer‡		Phenotype§		Virus titer†	log reduction in titer		Phenotype§
		Aman	Riman				Aman	Riman	
Clade 2.3.2.1c									
A/Cambodia/KSH230332/2023	9.9	1.3	1.8	Sensitive		7.6	2.0	2.2	Sensitive
A/Cambodia/NPH230032/2023, M2-S31N	8.8	0.2	0.0	Resistant		8.5	0.6	0.7	Resistant
Clade 2.3.4.4b									
A/Chile/25945/2023	10.1	0.5	0.7	*Resistant*		8.6	1.2	1.6	*Sensitive*
A/Texas/37/2024	10.1	1.1	1.7	Sensitive		8.4	3.6	5.5	Sensitive
A/Michigan/90/2024	NT	NT	NT	NT		7.0	5.1	5.1	Sensitive
Control viruses									
A/bald eagle/FL/2022 (H5N1)¶	9.2	2.2	2.3	Sensitive		7.0	3.4	3.5	Sensitive
A/Wisconsin/53/2009 (H1N1)pdm09#	7.6	3.0	3.1	Sensitive		6.6	3.0	3.7	Sensitive
A/California/07/2009 (H1N1)pdm09, M2-S31N#	5.8	0.0	0.2	Resistant		4.8	0.2	0.5	Resistant

*Italics indicate differences in phenotypes. Aman, amantadine; hpi, hours postinfection; NT, not tested; riman, rimantadine; TCID_50_, 50% tissue-culture infectious dose. †log_10_ TCID_50_/mL. ‡Maximum reduction in titer observed at M2 blocker concentrations <1 µg/mL. §Virus yields were determined in MDCK-SIAT1 cell culture supernatants collected at 21 or 16 hpi. Data shown are the average of 4 replicates from 2 independent experiments. Resistance to M2 blockers is defined as <1 log_10_ reduction in infectious virus yield at drug concentration <1 µg/mL. Concentration range of the M2 blockers used was 100–1,600 ng/mL. ¶Clade 2.3.4.4b influenza A(H5N1) virus, A/bald eagle/Florida/W22–134-OP/2022 (GISAID identification no. EPI_ISL_15063846; https://www.gisaid.org), was used as a control virus. #Control seasonal influenza A(H1N1)pdm09 viruses with or without the resistance marker M2-S31N.

**Table 2:** Susceptibility of highly pathogenic avian influenza A(H5N1) viruses isolated from humans to M2 blockers in cell culture–based IRINA, 2023–2024*

Influenza A(H5N1) virus	Differences in M2 protein sequence														Mean EC_50_ ±SD, ng/mL		GISAID ID
	12	13	14	18	28	31	50	51	61	82	88	89	95		Aman	Riman	
Clade 2.3.4.4b																	
A/bald eagle/FL/2022†	K	N	G	N	I	S	C	V	G	S	D	G	E		30.22 ± 13.33	8.36 ± 1.99	EPI_ISL_15063846
A/Chile/25945/2023‡	.	.	.	.	.	.	.	.	.	.	.	.	.		95.43 ± 11.69	16.70 ± 6.71	EPI_ISL_17468386
A/Chile/25945/2023 clone‡	.	.	.	.	.	.	.	.	.	.	.	.	.		26.73 ± 3.24	6.90 ± 0.52	NA
A/Texas/37/2024	.	.	.	.	.	.	.	.	.	.	N	.	.		24.11 ± 5.94	6.57 ± 1.71	EPI_ISL_19027114
A/Michigan/90/2024	.	.	.	.	.	.	.	.	.	.	N	.	.		29.60 ± 4.52	8.06 ± 1.54	EPI_ISL_19162802
A/Colorado/109/2024	.	.	.	.	.	.	.	.	R	.	N	.	.		14.88 ± 3.17	4.22 ± 0.02	EPI_ISL_19263923
A/Colorado/134/2024	.	.	.	.	.	.	.	.	R	.	N	.	.		NT	NT	EPI_ISL_19280426
A/Colorado/137/2024	.	.	.	.	.	.	.	.	.	.	N	.	.		25.21 ± 3.00	6.61 ± 0.57	EPI_ISL_19294963
A/Colorado/138/2024	.	.	.	.	.	.	.	.	.	.	N	.	.		NT	NT	EPI_ISL_19294962
A/Colorado/139/2024	.	.	.	.	.	.	.	.	.	.	N	.	.		17.65 ± 4.39	5.30 ± 0.69	EPI_ISL_19294964
A/Missouri/121/2024	.	.	.	.	.	.	.	.	.	.	N	.	.		VNR	VNR	EPI_ISL_19413343
A/California/134/2024	.	.	.	.	.	.	.	.	.	.	N	.	.		14.14 ± 3.07	4.28 ± 0.60	EPI_ISL_19463619
Clade 2.3.2.1c																	
A/Cambodia/NPH230032/2023§	R	K	E	R	V	**N**	Y	I	R	N	.	S	.		>1,000	>1,000	EPI_ISL_17024123
A/Cambodia/2302009/2023§	R	K	E	R	V	**N**	Y	I	R	N	.	S	.		>1,000	>1,000	EPI_ISL_17069010
A/Cambodia/NPH230776/2023	.	.	.	.	.	.	.	.	R	.	.	.	.		43.27 ± 10.46	12.27 ± 7.79	EPI_ISL_18373263
A/Cambodia/2310209/2023	.	.	.	.	.	.	.	.	R	.	.	.	.		NT	NT	EPI_ISL_18366401
A/Cambodia/KSH230332/2023	.	.	.	.	.	.	.	.	R	.	.	.	.		41.89 ± 13.31	9.84 ± 2.28	EPI_ISL_18543355
A/Cambodia/2311257/2023	.	.	.	.	.	.	.	.	R	.	.	.	K		59.73 ± 16.49	12.97 ± 1.74	EPI_ISL_18543643
A/Cambodia/24020155/2024	.	.	.	.	.	.	.	.	R	.	.	.	.		32.36 ± 10.13	6.48 ± 1.88	EPI_ISL_19270605
A/Cambodia/24020179/2024	.	.	.	.	.	.	.	.	R	.	.	.	.		34.73 ± 15.95	7.25 ± 2.96	EPI_ISL_19270607
A/Cambodia/SVH240441/2024	.	.	.	.	.	.	.	.	R	.	.	.	.		NT	NT	EPI_ISL_19312044
Control viruses¶																	
A/Wisconsin/53/2009 (H1N1)pdm09	R	S	E	R	.	.	.	I	R	.	.	.	.		25.49 ± 4.98	6.48 ± 1.46	EPI_ISL_63269
A/California/07/2009 (H1N1)pdm09	R	S	E	R	.	**N**	.	I	R	.	.	.	.		>1,000	>1,000	EPI_ISL_203615

*Data shown are means (+SDs) of >3 experiments. Concentration range of the M2 blockers used was ≈3.9–1,000 ng/mL. Dots indicate same amino acid residue as in A/bald eagle/Florida/W22-134-OP/2022. Amino acid substitutions at residues L26, V27, A30, S31, and G34 were implicated in resistance to M2-blockers. Aman, amantadine; EC_50_, 50% effective concentration; ID, identification; IRINA, influenza replication inhibition neuraminidase-based assay; NA, not applicable; NT, not tested; riman, rimantadine; VNR, virus not recovered. †Clade 2.3.4.4b influenza A(H5N1) virus, A/bald eagle/Florida/W22–134-OP/2022 (GISAID ID no. EPI_ISL_15063846; https://www.gisaid.org), was used as control. ‡See Appendix 1 Table 2). §Viruses collected from 2 family members in early 2023 that contain the M2-S31N resistance marker (in **bold**). ¶The M2 protein of the A/bald eagle/Florida/W22–134-OP/2022 differs from the control seasonal influenza A(H1N1)pdm09 virus by an additional 3 amino acid and A(H3N2) virus by an additional 10–11 amino acids.

To confirm the sequence-based assessment of drug phenotype, we tested a subset of those viruses by virus yield reduction assay. We included seasonal pH1N1 viruses with and without M2-S31N and an HPAI H5N1 virus, eagle/FL/22, as controls. At 21 hpi, yields of H5N1 viruses were 8.8–10.1 log_10_ TCID_50_/mL, higher than for seasonal viruses, 5.8–7.6 log_10_ TCID_50_/mL (Table 1). The criterion for sensitivity to M2 blockers was a reduction in virus yield at 1 µg/mL drug concentration, the highest physiologically achievable concentration (*34*). In the presence of amantadine and rimantadine, the drug-sensitive pH1N1 virus showed ≈3.0 log reduction, whereas its counterpart with M2-S31N demonstrated a <0.2 log reduction. A/Cambodia/NPH230032/2023 with M2-S31N also showed only <0.2 log reduction. A/Texas/37/2024 and A/Cambodia/KHS230332/2023 showed 1.1–1.8 log reductions consistent with a drug-sensitive phenotype. On the basis of those results, viruses in this study were identified as resistant when reduction in the viral yield was <1 log at drug concentrations below 1 µg/mL. Although Chile/23 and eagle/FL/22 shared the same M2 protein sequence, eagle/FL/22 showed greater reductions (≈2.3 log vs. 0.5–0.7 log) (Table 1). Testing was repeated by using a shorter replication time, 16 hpi, in which all viruses produced lower yields (Table 1). Except Chile/23, all tested viruses demonstrated from 2.0–5.1 log reductions in the presence of amantadine and from 2.2–5.5 log reductions in the presence of rimantadine, confirming their drug-sensitivity. At 16 hpi, Chile/23 demonstrated from 1.2–1.6 log reductions, which met the definition of a drug-sensitive phenotype as defined in this study (Table 1).

We next used IRINA, in which virus replication is limited to a single cycle, to assess M2 blocker susceptibility (Table 2). At 7 hpi, the 3 viruses with M2-S31N showed EC_50_s >1,000 ng/mL, whereas other viruses had EC_50_s ranging from 14 to 95 ng/mL for amantadine and 4 to 17 ng/mL for rimantadine. Chile/23 demonstrated elevated EC_50_s, which were ≈3-fold higher than for eagle/FL/22 (Table 2). Close inspection of NGS data for the Chile/23 isolate that we used for testing failed to show any virus subpopulations harboring M2 blocker resistance-conferring mutations. However, there was evidence of virus subpopulations with substitutions in HA, M1, PA, or PB1 proteins (Appendix 1 Table 2). Two substitutions (M1-A227S and PA-V91M) were present in both the clinical specimen and the isolate, whereas the other 2 substitutions (HA-N182S and PB1-K269Q) were not. The Chile/23 isolate was then used for virus purification by limiting dilution in cell culture. The resulting virus clone had the same consensus sequence as the virus in the original clinical specimen, and only minor subpopulations (6.7%–10.4%) were detected in PB1, PB2, and HA (Appendix 1 Table 2). In IRINA, the clone showed EC_50_s of 27 for amantadine and 7 ng/mL for rimantadine, consistent with a drug-sensitive phenotype (Table 2).

Overall, testing outcomes of the yield reduction assay at 16 hpi and IRINA agreed. In both assays, rimantadine was somewhat more active than amantadine at inhibiting replication of viruses lacking M2-S31N.

### Susceptibility to NA Inhibitors

The NAs of the 2 H5N1 clades differ by >50 amino acids, including a 20 amino acid deletion in the NA stalk of clade 2.3.2.1c (Appendix 1 Table 3). Sequence analysis did not identify molecular markers associated with reduced inhibition by NA inhibitors (*23*). The 2 early 2023 clade 2.3.2.1c viruses shared NA-V149I, a substitution near the NA active site that does not affect the susceptibility of clade 2.3.4.4b to NA inhibitors (*22*). However, the substitution’s effect on clade 2.3.2.1c viruses is unknown.

We tested all available isolates of H5N1 viruses in NA inhibition (NI) assay, including those with the NA-V149I substitution. NA inhibitors efficiently inhibited the NA enzyme activity of all viruses with IC_50_ values in the subnanomolar to low nanomolar ranges, supporting viruses’ susceptibility to oseltamivir, zanamivir, peramivir, laninamivir, and AV5080 (Table 3). Oseltamivir was least active at inhibiting the NA activity of viruses from both clades (0.74–3.99 nM IC_50_s), whereas AV5080 was most active (0.03–0.08 nM IC_50_s). We observed no differences in IC_50_s between the 2 clades, except for oseltamivir, which was ≈4-fold less active at inhibiting the NA activity of clade 2.3.4.4b viruses compared with clade 2.3.2.1c (3.61 vs. 0.96 nM median IC_50_) (Table 3).

**Table 3:** NA inhibitor susceptibility of highly pathogenic avian influenza A(H5N1) viruses isolated from humans in fluorescent NA inhibition assay, 2023–2024 *

Influenza A(H5N1) virus	Mean IC_50_, nM (fold change)					
	Oseltamivir	Zanamivir	Peramivir	Laninamivir	AV5080
Clade 2.3.2.1c, median IC_50_, n = 7	0.96	0.18	0.10	0.18	0.07
A/Cambodia/NPH230032/2023	0.78 ± 0.18	0.20 ± 0.05	0.08 ± 0.02	0.12 ± 0.03	0.05 ± 0.02
A/Cambodia/2302009/2023	1.00 ± 0.09	0.24 ± 0.04	0.09 ± 0.03	0.16 ± 0.03	0.05 ± 0.02
A/Cambodia/NPH230776/2023	0.74 ± 0.16	0.17 ± 0.03	0.11 ± 0.03	0.18 ± 0.04	0.07 ± 0.01
A/Cambodia/KSH230332/2023	1.03 ± 0.25	0.18 ± 0.02	0.12 ± 0.02	0.21 ± 0.04	0.08 ± 0.02
A/Cambodia/2311257/2023	0.99 ± 0.40	0.18 ± 0.02	0.10 ± 0.02	0.18 ± 0.02	0.07 ± 0.00
A/Cambodia/24020155/2024	0.96 ± 0.16	0.20 ± 0.01	0.10 ± 0.02	0.17 ± 0.02	0.06 ± 0.01
A/Cambodia/24020179/2024	0.90 ± 0.13	0.18 ± 0.02	0.10 ± 0.01	0.17 ± 0.01	0.06 ± 0.01
Clade 2.3.4.4b, median IC_50_, n = 7	3.61	0.20	0.08	0.16	0.04
A/Chile/25945/2023	2.98 ± 0.53	0.20 ± 0.02	0.09 ± 0.01	0.19 ± 0.03	0.04 ± 0.01
A/Texas/37/2024	3.16 ± 0.62	0.22 ± 0.03	0.10 ± 0.03	0.19 ± 0.03	0.04 ± 0.01
A/Michigan/90/2024	3.65 ± 0.71	0.19 ± 0.03	0.08 ± 0.02	0.16 ± 0.04	0.04 ± 0.01
A/Colorado/109/2024	3.99 ± 0.15	0.20 ± 0.02	0.08 ± 0.01	0.17 ± 0.03	0.03 ± 0.01
A/Colorado/137/2024	3.80 ± 0.15	0.19 ± 0.06	0.07 ± 0.00	0.16 ± 0.02	0.03 ± 0.00
A/Colorado/139/2024	3.51 ± 0.72	0.18 ± 0.03	0.07 ± 0.01	0.16 ± 0.03	0.03 ± 0.00
A/California/134/2024	3.61 ± 0.52	0.21 ± 0.02	0.07 ± 0.01	0.16 ± 0.02	0.04 ± 0.01
Control viruses†					
A/bald eagle/FL/2022 (H5N1)‡	3.07 ± 0.64	0.20 ± 0.04	0.09 ± 0.02	0.17 ± 0.03	0.04 ± 0.01
A/Illinois/45/2019 (H1N1)pdm09	0.34 ± 0.10	0.16 ± 0.04	0.05 ± 0.01	0.17 ± 0.01	0.07 ± 0.01
A/Alabama/03/2020 (H1N1)pdm09, NA-H275Y	201.27 ± 44.79 (592)	0.25 ± 0.06 (2)	15.80 ± 2.74 (316)	0.38 ± 0.04 (2)	0.78 ± 0.14 (11)
A/Pennsylvania/46/2015 (H3N2)	0.15 ± 0.01	0.22 ± 0.04	0.08 ± 0.01	0.35 ± 0.08	0.27 ± 0.04
A/Washington/33/2014 (H3N2), NA-E119V	47.33 ± 6.18 (315)	0.42 ± 0.05 (2)	0.11 ± 0.01 (1)	0.48 ± 0.09 (1)	0.16 ± 0.03 (1)
B/North Carolina/25/2018 (Vic)	23.70 ± 1.83	1.97 ± 0.63	0.55 ± 0.07	1.64 ± 0.13	0.87 ± 0.12
B/Missouri/12/2018 (Vic), NA-D197E	165.21 ± 20.39 (7)	13.12 ± 4.95 (7)	8.01 ± 0.3.11 (15)	5.20 ± 2.27 (3)	2.20 ± 0.52 (3)

*Data shown are means +SDs of >3 experiments. Fold change was calculated from the subtype sequence-matched control virus. IC_50_, 50% inhibitory concentration; NA, neuraminidase. †Control seasonal influenza A viruses were from the Centers for Disease Control and Prevention Neuraminidase Inhibitor Susceptibility Reference Virus Panel International Reagent resource (no. FR-1755 ver3). ‡Clade 2.3.4.4b influenza A(H5N1) virus, A/bald eagle/Florida/W22–134-OP/2022 (GISAID ID no. EPI_ISL_15063846; https://www.gisaid.org), was used as a control.

### Susceptibility to Polymerase Inhibitors

Assessing susceptibility to PA inhibitors by sequence analysis revealed substantial amino acid differences within the endonuclease domain between the 2 H5N1 clades (Appendix 2 Table 1). Virus sequences did not contain molecular markers of known association with reduced baloxavir susceptibility (*23*). In IRINA, the EC_50_s of baloxavir (0.40–1.06 nM) and tivoxavir (0.43–1.09 nM) were low and similar to those of seasonal influenza A viruses (Table 4). Those results indicate that the clade 2.3.2.1c and 2.3.4.4b viruses tested were susceptible to both PA inhibitors with a similar susceptibility.

**Table 4:** Susceptibility of HPAI A(H5N1) viruses isolated from humans to polymerase inhibitors in cell culture-based assay IRINA, 2023–2024*

Influenza A(H5N1) virus	Mean EC_50_, nM			
	PA-CEN inhibitor			PB2 inhibitor
	Baloxavir (fold change)	Tivoxavir (fold change)		Pimodivir (fold change)
Clade 2.3.2.1c, median EC_50_, n = 7	0.55	0.52		0.40
A/Cambodia/NPH230032/2023	0.56 ± 0.20	0.84 ± 0.23		0.06 ± 0.01
A/Cambodia/2302009/2023	0.74 ± 0.25	0.43 ± 0.18		0.07 ± 0.02
A/Cambodia/NPH230776/2023	0.54 ± 0.08	0.56 ± 0.22		0.37 ± 0.10
A/Cambodia/KSH230332/2023	0.55 ± 0.12	0.52 ± 0.13		0.34 ± 0.14
A/Cambodia/2311257/2023	0.62 ± 0.12	0.54 ± 0.17		0.40 ± 0.09
A/Cambodia/24020155/2024	0.54 ± 0.16	0.52 ± 0.14		0.91 ± 0.31
A/Cambodia/24020179/2024	0.40 ± 0.14	0.47 ± 0.12		0.45 ± 0.06
Clade 2.3.4.4b, median EC_50_, n = 7	0.83	0.88		1.32
A/Chile/25945/2023	0.96 ± 0.28	1.07 ± 0.17		0.73 ± 0.18
A/Texas/37/2024	1.06 ± 0.22	1.09 ± 0.09		1.66 ± 0.17
A/Michigan/90/2024	0.57 ± 0.21	0.56 ± 0.22		1.48 ± 0.65
A/Colorado/109/2024	0.44 ± 0.35	0.72 ± 0.30		1.32 ± 0.46
A/Colorado/137/2024	0.95 ± 0.30	0.98 ± 0.24		1.51 ± 0.27
A/Colorado/139/2024	0.70 ± 0.07	0.77 ± 0.19		1.19 ± 0.12
A/California/134/2024	0.73 ± 0.12	0.47 ± 0.07		1.03 ± 0.30
Control viruses†				
A/bald eagle/FL/2022 (H5N1)‡	0.42 ± 0.20	0.76 ± 0.29		0.46 ± 0.19
A/Illinois/08/2018 (H1N1)pdm09	1.16 ± 0.24	1.46 ± 0.32		0.93 ± 0.22
A/Illinois/08/2018 (H1N1)pdm09, PA-I38T	137.05 ± 17.43 (116)	72.86 ± 11.00 (50)		0.49 ± 0.14
A/New Jersey/24/2017 (H3N2)	0.72 ± 0.14	0.57 ± 0.26		0.60 ± 0.25
A/Pennsylvania/242/2017 (H3N2), PB2-S342R	0.35 ± 0.10	0.29 ± 0.11		99.50 ± 27.26 (166)

*Data shown are means +SDs of >3 experiments. Fold change was calculated from sequence-matched control virus. EC_50_, 50% effective concentration; HPAI, highly pathogenic avian influenza; IRINA, influenza replication inhibition neuraminidase-based assay; PA-CEN, PA cap-dependent endonuclease. †Control seasonal viruses were from Centers for Disease Control and Prevention Baloxavir Susceptibility Reference Virus Panel (no. FR-1678 ver1.1) and virus inventory. ‡Clade 2.3.4.4b A(H5N1) virus, A/bald eagle/Florida/W22–134-OP/2022 (GISAID ID no. EPI_ISL_15063846), was used as a control.

None of the study viruses contained reported markers of pimodivir resistance (Appendix 2 Table 2) (*27*,*35*). Pimodivir effectively inhibited all viruses with EC_50_s in a subnanomolar range (Table 4). Pimodivir EC_50_s of the early 2023 clade 2.3.2.1c viruses were ≈6-fold lower compared with other viruses from this clade, which contained internal genes mostly from clade 2.3.4.4.b viruses, except for nucleoprotein. Altogether, those results highlight a potent in vitro antiviral effect by pimodivir against H5N1 viruses from both clades.

## Discussion

Our study shows HPAI H5N1 clade 2.3.2.1c and clade 2.3.4.4b viruses isolated from sporadic human cases in Cambodia, Chile, and the United States during 2023–2024 are susceptible to approved NA inhibitors and the PA inhibitor baloxavir. The viruses were also susceptible to M2 blockers, except for the 2 nonreassortant viruses isolated in Cambodia during 2023. Viruses from both clades were susceptible to investigational antivirals AV5080, which targets viral segment NA; tivoxavir, which targets viral segment PA; and pimodivir, which targets viral segment PB2.

Nearly all seasonal influenza viruses that have circulated since 2010 were resistant to M2 blockers. In addition, M2 blocker resistance was seen in certain groups of swine and avian influenza A viruses (*18*,*19*), which greatly reduced the appeal of this inexpensive class of oral antivirals. However, they may remain useful in certain instances, such as controlling zoonotic outbreaks caused by drug-sensitive viruses, especially when administered in combination with other antivirals (*36*). Combined therapy may produce a synergistic antiviral effect leading to substantial reduction of viral titers thus lowering the risk for resistance emergence and speeding up recovery (*16*).

In yield reduction assay, the testing outcome for Chile/23 was inconclusive because the criterion for susceptibility to M2 blockers was met at 16hpi, but not at 21hpi. Some of the HPAI H5N1 viruses tested in this study had mammalian-adaptive molecular signatures in their PB2 protein (i.e., Q591K, E627K, M631L, D701N) (Appendix 2 Table 2) (*37*,*38*). Regardless, all H5N1 viruses grew to high yields in a mammalian cell line. To address concerns over the effect of different virus replication kinetics on testing outcomes, we used the new assay, IRINA, for the first time to assess M2 blocker susceptibility as it is based on a single-cycle replication (*33*). Apart from providing improved throughput and turnaround time compared with the traditional assays, IRINA enabled more definitive identification of drug-resistant viruses whose EC_50_s were >1,000 ng/mL. Drug-sensitive viruses showed EC_50_s <100 ng/mL for amantadine and <20 ng/mL for rimantadine. Compared with eagle/FL/22, Chile/23 was less susceptible to M2 blockers in both yield reduction assay and IRINA, despite having the same M2 sequence. On the other hand, the Chile/23 clone, whose genomic sequence showed only minor virus subpopulations in viral segments other than M2 had the same EC_50_s as eagle/FL/22 in IRINA. Additional studies are underway to investigate molecular mechanisms underlying the decreased susceptibility of the Chile/23 isolate.

The NA sequences of clades 2.3.2.1c and 2.3.4.4b differ substantially in the stalk and the head region (Appendix 1 Table 3). Regardless of virus or NA inhibitor, IC_50_s fell within subnanomolar ranges, and thus, all H5N1 viruses in this study were deemed susceptible to this class of antiviral drugs. However, we found that oseltamivir was ≈4-fold more active at inhibiting the NA activity of clade 2.3.2.1c than clade 2.3.4.4b viruses (Table 3). Differences in oseltamivir IC_50_s among various clades of H5N1 viruses have been previously reported (*39*–*42*). Binding of oseltamivir within the NA active site involves a side chain reorientation at residue E277, and differences at residue 253 and other neighboring amino acids can lead to steric effects that elevate oseltamivir IC_50_s (*43*). It is also known that temperature, substrate, buffer pH, and other experimental conditions can affect NI testing outcomes (*32*). Taking those into consideration, uniform temperature was maintained during NI testing by incubating each microplate in a single file. Of interest, clade 2.3.2.1a (from Bangladesh) and 2.3.4.4b (from the United States) viruses were recently reported to show the same oseltamivir IC_50_s despite substantial difference in NA sequences (up to 31 aa) (*27*). Directly comparing results generated by different laboratories is challenging because it requires including the same reference viruses for comparison. Therefore, we used Centers for Disease Control and Prevention reference virus panels (https://www.internationalreagentresource.org), which are available to laboratories conducting antiviral surveillance.

The EC_50_s for the PA inhibitors baloxavir and tivoxavir, an investigational drug undergoing phase 1 clinical trials (*25*), were in a low nanomolar range for H5N1 viruses. We also demonstrated that viruses from both clades were susceptible to the PB2 inhibitor pimodivir. Two clade 2.3.2.1c viruses collected in early 2023 displayed the highest pimodivir susceptibility. Those viruses share a PB2-V356I substitution, which is flanked by residues R355 and H357 involved in pimodivir binding (*26*). Therefore, our study provides evidence for the value of PB2 inhibitors as additional options to control influenza A virus infections (*26*), pending drug structure refinement and further investigation.

Our study limitations included laboratory data interpretation. For M2 blockers, resistant viruses are identified on the basis of their diminished replication in cell culture at a specific drug concentration of 1,000 ng/mL (*34*). Conversely, cell culture-based assays cannot be used to predict susceptibility of viruses to NA inhibitors. Moreover, there are no concentration-based criteria to identify NA inhibitor-resistant viruses by using a functional NI assay. Instead, antiviral testing in surveillance identifies outliers (viruses with IC_50_s above the subtype or lineage-specific baseline) and reports them as exhibiting reduced (10–100-fold) or highly reduced (>100-fold) inhibition of NA enzyme activity. However, this approach is challenging when reporting results for zoonotic viruses that are more genetically diverse than seasonal viruses. For surveillance purposes, antiviral susceptibility testing should be conducted if viral genomic changes are detected, whether by evolution, selection, or host adaptation, because that may affect the ability of antivirals to interfere with the function of the targeted viral proteins. By using an in vitro approach, we demonstrated there are no such changes in clade 2.3.2.1c and 2.3.4.4b H5N1 viruses isolated from humans in 2023 and 2024 in this study, except the early 2023 viruses from Cambodia. However, laboratory results should be interpreted with caution because variables such as virus virulence, time of treatment initiation, patient immune status, and other factors can affect the outcome of antiviral treatment. For example, no correlation was observed between IC_50_s and oseltamivir treatment outcomes in mice infected with HPAI H5N1 viruses (*39*).

In recent years, monitoring systems have sporadically detected oseltamivir- and baloxavir-resistant H5N1 viruses in wild birds, including in clade 2.3.4.4b (*22*,*25*,*44*). Drug-resistant influenza viruses may emerge following treatment, especially in young children and immunocompromised patients (*45*). Although drug-resistant influenza viruses often show impaired replicative fitness, the concern is their ability to gain a selective advantage because of reassortment and continuous evolution. Hence, new antiviral drugs, including those with novel mechanisms of action, and their combinations, would be a welcome addition to the current antiinfluenza arsenal. Compared with monotherapy, combination treatment potently inhibits H5N1 virus replication and improves survival rates in mice (*36*,*46*,*47*). Data are needed on higher oseltamivir dosing and combination antiviral treatment of patients infected with recent H5N1 viruses to inform treatment recommendations. 

In conclusion, although the clinical translation of laboratory findings remains to be seen, our data do not change current recommendations to initiate oseltamivir treatment as soon as possible for patients with confirmed or suspected H5N1 (*48*), and postexposure prophylaxis of close contacts of H5N1 cases (*49*). However, higher antiviral dosing and combination antiviral treatment (e.g., oseltamivir and baloxavir) should be considered, in particular for patients with H5N1 who are hospitalized or immunocompromised.

Appendix 1 Additional information about antiviral susceptibility of clade 2.3.2.1c and 2.3.4.4b H5N1 viruses from humans, 2023–2024

Appendix 2 Additional information about amino acid differences between clade 2.3.2.1c and 2.3.4.4b H5N1 viruses from humans, 2023–2024

## Figures and Tables

**Table:** 



