# Supplementary material for: Antiviral Susceptibility of Influenza A(H5N1) Clade 2.3.2.1c and 2.3.4.4b Viruses from Humans, 2023–2024
Source: Emerg Infect Dis. 2025 Apr;31(4):751–60. doi: 10.3201/eid3104.241820 (PMC11950254; doi:10.3201/eid3104.241820)
Supplement: Appendix 1 — Additional information about antiviral susceptibility of clade 2.3.2.1c and 2.3.4.4b H5N1 viruses from humans, 2023–2024 [file 24-1820-Techapp-s1.pdf]

Article DOI: https://doi.org/10.3201/eid3104.241820

EID cannot ensure accessibility for supplementary materials supplied by authors. Readers who have difficulty accessing supplementary content should contact the authors for assistance.

Antiviral Susceptibility of Influenza A(H5N1) Clade 2.3.2.1c and 2.3.4.4b Viruses from Humans, 2023–2024

Appendix 1

Material and Methods

Antiviral compounds

The M2-blockers amantadine hydrochloride (Sigma-Aldrich) and rimantadine (Hoffman-La Roche) were diluted in distilled water to give 2 mg/mL. NA-inhibitors oseltamivir carboxylate (oseltamivir), zanamivir, peramivir, laninamivir (BioSynth), and AV5080 (provided by ChemDiv) were prepared in 20 mM stocks by dissolving in distilled water. The PA-inhibitors baloxavir acid (baloxavir, MedChem Express) and tivoxavir (AV5116, provided by ChemDiv), and PB2-inhibitor pimodivir (MedChemExpress) were dissolved in DMSO as 1 mM stocks. Antivirals were stored at −20°C until used and were serially diluted in appropriate assay buffer or virus growth medium when used in respective assays.

Viruses

HPAI A(H5N1) viruses were submitted by U.S. public health laboratories and by National Influenza Centers in Chile and Cambodia to CDC, Atlanta, GA. HPAI A(H5N1) virus A/bald eagle/Florida/W22–134-OP/2022 (GISAID ID EPI_ISL_15063846) was provided by USDA (*1*). Viruses were propagated in 10-day old embryonated chicken eggs or in Madin-Darby canine kidney (MDCK) cells (ATCC, Manassas, VA). The CDC Antiviral Susceptibility Reference Virus Panels (FR-1755 ver3 and FR-1678 ver1.1; International Reagent Resource (https://www.internationalreagentresource.org) were used as controls in phenotypic assays. Seasonal viruses with markers of M2 blocker (M2-S31N) or pimodivir (PB2-S342R) resistance and their corresponding sequence-matched control viruses were from the CDC virus repository.

Handling and experiments with HPAI A(H5N1) viruses were conducted in an enhanced biosafety level 3 containment facility.

Next generation sequencing analysis

Influenza genome sequences were generated using the next generation sequencing (NGS) (Illumina), analyzed by the iterative refinement meta-assembler (IRMA) (*2*) and were aligned with sequences available from the Global Initiative on Sharing All Influenza Data (GISAID) using MAFFT ver7 (*3*). All sequences were deposited in public databases **(Appendix Table 1).**

Virus yield reduction

A subset of A(H5N1) viruses was tested in a conventional yield reduction assay (*6*) to assess virus susceptibility to M2-blockers with minor modifications. Briefly, confluent monolayers of MDCK-SIAT1 cells were inoculated with virus (multiplicity of infection = 0.001 or 0.004) and growth medium containing either amantadine or rimantadine (concentration range: 100–1600 ng/mL) was added followed by incubation at 37°C; TPCK-treated trypsin was added to growth medium for seasonal control viruses. After 16- or 21-hour post-infection (hpi), supernatants were collected, and virus yields were determined in MDCK-SIAT1 cells in the presence of TPCK-treated trypsin. The collection time points were arbitrarily selected to accommodate the faster growth kinetics of A(H5N1) viruses and its effect on testing outcome. Virus yields were expressed as a 50% tissue-culture infectious dose ($TCID_{50}$)/mL according to Spearman-Kaerber method.

Neuraminidase Inhibition (NI) assay

Virus susceptibility to NA-inhibitors was assessed using the NA-Fluor Influenza Neuraminidase Assay Kit (Applied Biosystems) in 96-well black microplates as previously described (*7*). Normalized virus preparations were pre-incubated with the NA inhibitors for 45 mins, followed by 1h incubation with the 2-(4-(methylumbelliferyl)-a-D-N-acetylneuraminic acid (MUNANA, Sigma) substrate; fluorescence was measured using Cytation 7 (Agilent-Biotek). To ensure uniform temperature, each microplate was incubated individually. The IC_{50} (the drug concentration required to inhibit 50% NA activity) was calculated based on at least three independent tests.

Influenza replication inhibition neuraminidase-based assay (IRINA) to assess susceptibility to M2 blockers and polymerase inhibitors

Susceptibility to M2-blockers and polymerase inhibitors was assessed using the cell-culture based assay IRINA as previously described (*6*). MDCK-SIAT1 cell suspension was added to wells (0.7×10^5 cells/well) of a 96-well microplate (Agilent) containing serially diluted antiviral and a normalized virus preparation (to produce NA activity on surface of infected cells that is equivalent to 1500 pmol/well of 4-methylumbelliferone). Microplates were then incubated at 37°C for 7h, after which, supernatants were aspirated and MUNANA was added to monolayer. After 1h incubation at 37°C, the reaction was stopped, and virus replication was assessed by measuring fluorescence (460 nm/360 nm, excitation/emission) that was read from the bottom using Cytation 7 (Agilent-BioTek). Relative fluorescent unit (RFU) readouts were plotted using nonlinear regression to determine 50% effective concentration (EC_{50}) values (*8*).

Appendix Table 1. Clade 2.3.2.1c and 2.3.4.4b highly pathogenic avian A(H5N1) viruses detected in humans in Cambodia, Chile, and United States from January 2023–September 2024*

Influenza A(H5N1) virus	Collection date	Animal exposure†	Disease severity, outcome	Genotype‡	GISAID ID (EPI_ISL_)	GISAID ID for gene segment (EPI)				Originating lab/submitter institution
						M	NA	PA	PB2	
Clade 2.3.2.1c										
A/Cambodia/NPH230032/2023§	23Feb21	poultry or bird	Critical, fatal	Non-reassortant	17024123	2419701	2419702	2419697	2419699	Pasteur, Phnom Penh, Cambodia
A/Cambodia/2302009/2023§	23Feb23	poultry or bird	Mild	Non-reassortant	17069010	2436450	2436454	2436462	2436466	Pasteur, Phnom Penh, Cambodia
A/Cambodia/NPH230776/2023	23Oct06	poultry or bird	Critical, fatal	Reassortant	18373263	2771060	2771069	2771062	2771065	NIH PL, Phnom Penh, Cambodia
A/Cambodia/2310209/2023	23Oct07	poultry or bird	Critical, fatal	Reassortant	18366401	2767577	2767581	2767578	2767579	NIH PL, Phnom Penh, Cambodia
A/Cambodia/KSH230332/2023	23Nov23	poultry or bird	Critical, fatal	Reassortant	18543355	2804250	2804249	2804246	2804244	NIH PL, Phnom Penh, Cambodia
A/Cambodia/2311257/2023	23Nov24	poultry or bird	Mild	Reassortant	18543643	2804269	2804268	2804265	2804263	NIH PL, Phnom Penh, Cambodia
A/Cambodia/i0125001G/2024	24Jan23	poultry or bird	Severe, fatal	Reassortant	18823967	-	2960108	-	-	NIH PL, Phnom Penh, Cambodia
A/Cambodia/24020155/2024	24Feb08	poultry or bird	Asymptomatic	Reassortant	19270605	3442276	3442280	3442277	3442278	NIH PL, Phnom Penh, Cambodia/CDC-Atlanta
A/Cambodia/24020179/2024	24Feb10	poultry or bird	Mild	Reassortant	19270607	3442285	3442293	3442286	3442287	NIH PL, Phnom Penh, Cambodia/CDC-Atlanta
A/Cambodia/24070331/2024	24Jul30	poultry or bird	Mild	Reassortant	19312043	-	-	-	-	Pasteur, Phnom Penh, Cambodia
A/Cambodia/SVH240441/2024	24Aug02	poultry or bird	Critical, survived	Reassortant	19312044	-	3489220	-	-	Pasteur, Phnom Penh, Cambodia
A/Cambodia/KSH240409/2024	24Aug17	poultry or bird	Critical, fatal	Reassortant	19353003	-	-	-	-	Pasteur, Phnom Penh, Cambodia
Clade 2.3.4.4b										
A/Chile/25945/2023	23Mar24	Unknown	Critical, survived	B3.2	17468386	2510186	2510185	2510182	2510180	Instituto de Salud Publica de Chile
A/Texas/37/2024	24Mar28	dairy cows	Mild	B3.13	19027114	3171493	3171486	3171487	3171492	Texas DSHS/CDC-Atlanta
A/Michigan/90/2024	24May14	dairy cows	Mild	B3.13	19162802	3334177	3334181	3334178	3334179	Michigan DCH/CDC-Atlanta
A/Michigan/91/2024	24May25	dairy cows	Mild	B3.13	19177746	-	3352528	-	-	Michigan DCH/CDC-Atlanta
A/Colorado/108/2024	24Jun28	dairy cows	Mild	-	VNR	-	-	-	-	Colorado DHL/CDC-Atlanta
A/Colorado/109/2024	24Jul11	poultry	Mild	B3.13	19263923	3437001	3437005	3437002	3437003	Colorado DHL/CDC-Atlanta
A/Colorado/110/2024	24Jul11	poultry	Mild	-	VNR	-	-	-	-	Colorado DHL/CDC-Atlanta

Influenza A(H5N1) virus	Collection date	Animal exposure†	Disease severity, outcome	Genotype‡	GISAID ID (EPI_ISL_)	GISAID ID for gene segment (EPI) M	NA	PA	PB2	Originating lab/submitter institution
A/Colorado/111/2024	24Jul11	poultry	Mild	-	VNR	-	-	-	-	Colorado DHL/CDC-Atlanta
A/Colorado/112/2024	24Jul11	poultry	Mild	-	VNR	-	-	-	-	Colorado DHL/CDC-Atlanta
A/Colorado/124/2024	24Jul11	poultry	Mild	-	VNR	-	-	-	-	Colorado DHL/CDC-Atlanta
A/Colorado/134/2024	24Jul15	poultry	Mild	B3.13	19280426	3452196	3452198	-	3452197	Colorado DHL/CDC-Atlanta
A/Colorado/137/2024	24Jul19	poultry	Mild	B3.13	19294963	3467508	3467512	3467509	3467510	Colorado DHL/CDC-Atlanta
A/Colorado/138/2024	24Jul19	poultry	Mild	B3.13	19294962	3467500	3467504	3467501	3467502	Colorado DHL/CDC-Atlanta
A/Colorado/139/2024	24Jul22	poultry	Mild	B3.13	19294964	3467516	3467520	3467517	3467518	Colorado DHL/CDC-Atlanta
A/Missouri/121/2024	24Aug22	Unknown	Severe, survived	-	19413343	3556414	3556415	-	-	Missouri DHSS/CDC-Atlanta
A/California/134/2024	24Sep30	dairy cows	Mild	B3.13	19463619	-	3591635	-	-	California DHS/CDC-Atlanta

*DCH, Department of Community Health; DHL, Department of Health Lab; DHSS, Department of Health, and Senior Service; DSHS, Department of State Health Services; NIH PL, National Institute of Public Health; n/a, not applicable; Pasteur, Institut Pasteur du Cambodia; VNR, virus not recovered.
†Animal exposure, patients were exposed to infected or sick animal.
‡Genotype based on USDA genotyping system in clade 2.3.4.4b virus for Chile and U.S. isolates (*4*) or reassortment between clade 2.3.2.1c and 2.3.4.4b viruses in Cambodia (*5*).
§Non-reassortant clade 2.3.2.1c viruses collected from two family members in early 2023.
Available M, NA, PA, and PB2 gene sequences of A(H5N1) viruses submitted by U.S. public health laboratories and by National Influenza Centers in Chile and Cambodia to CDC, Atlanta, GA. Additional sequences were downloaded from the Global Initiative on Sharing All Influenza Data (GISAID) public database (last accessed in September 2024). Sequences were compiled and analyzed using MAFFT version 7 program. Dash lines indicate no sequence available.

Appendix Table 2. Virus subpopulations in A/Chile/25945/2023 (H5N1) virus preparations

		Codon		Virus subpopulations		
Protein	Amino acid position	Consensus	Minority	Original specimen	Virus isolate (C2)	Clone (C2S1)
HA	73	GTG	ATG	V73M (23.6%)	-	-
	182	AAC	AAA	N182K (33.9%)	-	-
			AGC	-	N182S (5.3%)	-
	310	AAG	AAT	-	-	K310N (6.7%)
M1	227	GCC	TCC	A227S (10.7%)	A227S (40.7%)	-
PA	91	GTG	ATG	V91M (11.7%)	V91M (56.2%)	-
PB1	279	AAG	CAG	-	K279Q (7.9%)	K279Q (10.4%)
PB2	380	AGG	AAG	-	-	R380K (8.5%)

The consensus genome sequence of the A/Chile25945/2023 (H5N1) virus in the original specimen (GISAID ID no. EPI_ISL_17468386) was deposited by Instituto de Salud Publica de Chile. In our study, Illumina MiSeq was used to generate sequences that were analyzed using the IRMA approach with single nucleotide variant threshold of 5% (*2*). The minority allele is indicated by underline. Dash line (-), no detection of virus subpopulation (<5% threshold); C2, virus isolate passaged twice in MDCK cells; C2S1, clone was obtained using limiting dilution technique in MDCK-SIAT1 cells.

Appendix Table 3. Susceptibility of HPAI A(H5N1) viruses to NA inhibitors based on sequence analysis

Influenza A(H5N1) virus	GISAID ID (EPI_ISL_)	Stalk deletion*	Amino acid differences in NA (head domain)																					
			80	81	95	99	100	105	149	155	163	188	211	234	240	269	287	321	336	338	339	395	407	443
Clade 2.3.4.4b																								
A/bald eagle/FL/2022†	15063846	No	V	T	S	I	Y	G	V	Y	V	I	I	V	T	L	D	V	S	M	S	E	V	I
A/Chile/25945/2023	17468386	No	.	.	.	.	.	.	.	.	.	.	.	.	.	M	.	.	.	.	P	.	.	.
A/Texas/37/2024	19027114	No	.	.	.	.	.	.	.	.	.	.	.	.	.	M	.	I	.	.	P	.	.	.
A/Michigan/90/2024	19162802	No	.	.	.	.	.	.	.	.	.	.	.	.	.	M	.	I	.	.	P	.	.	.
A/Michigan/91/2024	19177746	No	.	.	.	.	.	.	.	.	.	.	.	.	.	M	.	I	.	.	P	.	.	.
A/Colorado/109/2024	19263923	No	.	.	.	.	.	.	.	.	.	.	.	.	.	M	.	I	.	.	P	.	.	.
A/Colorado/134/2024	19280426	No	.	.	.	.	.	.	.	.	.	.	.	.	.	M	.	I	.	.	P	.	.	.
A/Colorado/137/2024	19294963	No	.	.	.	.	.	.	.	.	.	.	.	.	.	M	.	I	.	.	P	.	.	.
A/Colorado/138/2024	19294962	No	.	.	.	.	.	.	.	.	.	.	.	.	.	M	.	I	.	.	P	.	.	.
A/Colorado/139/2024	19294964	No	.	.	.	.	.	.	.	.	.	.	.	.	.	M	.	I	.	.	P	.	.	.
A/Missouri/121/2024	19413343	No	.	.	.	.	.	.	.	.	.	.	.	.	.	M	.	I	.	.	P	.	F	.
A/California/134/2024	19463619	No		.	.	.	.	.	.	.	.	.	.	.	.	M	.	I	.	.	P	.	.	.
Clade 2.3.2.1c																								
A/Cambodia/NPH230032/2023‡	17024123	Yes	L	A	K	V	H	S	I	H	I	T	.	.	A	.	E	.	G	V	.	A	.	L
A/Cambodia/2302009/2023‡	17069010	Yes	L	A	K	V	H	S	I	H	I	T	.	.	A	.	E	.	G	V	.	A	.	L
A/Cambodia/NPH230776/2023	18373263	Yes	.	A	K	.	Q	S	.	H	I	T	L	.	A	.	E	.	G	V	.	A	.	L
A/Cambodia/2310209/2023	18366401	Yes	.	A	K	.	H	S	.	H	I	T	L	.	A	.	E	.	G	V	.	A	.	L
A/Cambodia/KSH230332/2023	18543355	Yes	.	A	K	.	H	S	.	H	I	T	L	.	A	.	E	.	G	V	.	A	.	L
A/Cambodia/2311257/2023	18543643	Yes	.	A	K	.	H	S	.	H	I	T	L	.	A	.	E	.	G	V	.	A	.	L
A/Cambodia/i0125001G/2024	18823967	Yes	.	A	K	.	H	S	.	H	I	T	L	.	A	.	E	.	G	V	.	A	.	L
A/Cambodia/24020155/2024	19270605	Yes	.	A	K	.	H	S	.	H	I	T	L	.	A	.	E	.	G	V	.	A	.	L
A/Cambodia/24020179/2024	19270607	Yes	.	A	K	.	H	S	.	H	I	T	L	.	A	.	E	.	G	V	.	A	.	L
A/Cambodia/SVH240441/2024	19312044	Yes	.	A	K	.	H	S	.	H	I	T	L	I	A	.	E	I	G	V	.	A	.	L

*Twenty amino acid stalk deletion at position 48–68.
†Clade 2.3.4.4b A(H5N1) virus, A/bald eagle/Florida/W22–134-OP/2022, was used for reference purposes.
‡Nonreassortant clade 2.3.2.1c viruses collected from 2 family members in early 2023 which contain the NA-V149I substitution.
Dots indicate same amino acid residue as in A/bald eagle/Florida/W22–134-OP/2022. IC_{50} values for viruses available for testing are shown in manuscript Table 3.

References

1. Nguyen HT, Chesnokov A, De La Cruz J, Pascua PNQ, Mishin VP, Jang Y, et al. Antiviral susceptibility of clade 2.3.4.4b highly pathogenic avian influenza A(H5N1) viruses isolated from birds and mammals in the United States, 2022. Antiviral Res. 2023;217:105679. PubMed https://doi.org/10.1016/j.antiviral.2023.105679

2. Shepard SS, Meno S, Bahl J, Wilson MM, Barnes J, Neuhaus E. Viral deep sequencing needs an adaptive approach: IRMA, the iterative refinement meta-assembler. BMC Genomics. 2016;17:708. PubMed https://doi.org/10.1186/s12864-016-3030-6

3. Katoh K, Standley DM. MAFFT multiple sequence alignment software version 7: improvements in performance and usability. Mol Biol Evol. 2013;30:772–80. PubMed https://doi.org/10.1093/molbev/mst010

4. Youk S, Torchetti MK, Lantz K, Lenoch JB, Killian ML, Leyson C, et al. H5N1 highly pathogenic avian influenza clade 2.3.4.4b in wild and domestic birds: Introductions into the United States and reassortments, December 2021-April 2022. Virology. 2023;587:109860. PubMed https://doi.org/10.1016/j.virol.2023.109860

5. Siegers JY, Xie R, Byrne AMP, Edwards KM, Hu S, Yann S, et al. Emergence of a Novel Reassortant Clade 2.3.2.1c Avian Influenza A/H5N1 Virus Associated with Human Cases in Cambodia. medRxiv. 2024;1101:24313747. https://doi.org/10.1101/2024.11.04.24313747

6. Gubareva LV, Trujillo AA, Okomo-Adhiambo M, Mishin VP, Deyde VM, Sleeman K, et al. Comprehensive assessment of 2009 pandemic influenza A (H1N1) virus drug susceptibility in vitro. Antivir Ther. 2010;15:1151–9. PubMed https://doi.org/10.3851/IMP1678

7. Okomo-Adhiambo M, Mishin VP, Sleeman K, Saguar E, Guevara H, Reisdorf E, et al. Standardizing the influenza neuraminidase inhibition assay among United States public health laboratories conducting virological surveillance. Antiviral Res. 2016;128:28–35. PubMed https://doi.org/10.1016/j.antiviral.2016.01.009

8. Patel MC, Flanigan D, Feng C, Chesnokov A, Nguyen HT, Elal AA, et al. An optimized cell-based assay to assess influenza virus replication by measuring neuraminidase activity and its applications for virological surveillance. Antiviral Res. 2022;208:105457. PubMed https://doi.org/10.1016/j.antiviral.2022.105457
